# Parents’ approaches to sexuality education of their adolescent boys: a qualitative study in Ahvaz, Iran

**DOI:** 10.1186/s12978-022-01367-0

**Published:** 2022-03-19

**Authors:** Zahra Rouhparvar, Mojgan Javadnoori, Shadab Shahali

**Affiliations:** 1 School of Nursing and Midwifery, Ahvaz Jundishapur University of Medical Sciences, Ahvaz, Iran; 2 Reproductive Health Promotion Research Center, Ahvaz Jundishapur University of Medical Sciences, Ahvaz, Iran; 3 Department of Reproductive Health and Midwifery, Faculty of Medical Sciences, Tarbiat Modares University, Tehran, Iran

**Keywords:** Sex education, Child rearing, Parents, Adolescent, Iran

## Abstract

**Background:**

Despite its important role in adolescent sexual health, sexuality education remains one of the most challenging responsibilities of families, especially those living in Muslim communities which experience the transition to modernity. There is little information about sexuality education of boys in Iran. This study aimed to explore parents’ approaches to sexuality education of adolescent boys in Ahvaz, southwest of Iran.

**Methods:**

This qualitative study was conducted in 2017 in Ahvaz, Iran. Participants were selected through purposeful sampling with maximum variation. Qualitative data were collected by conducting semi-structured in-depth interviews and focused group discussions with 27 parents from middle/high social class who had adolescent boys aged between 10 and 19. Data were analyzed using conventional qualitative content analysis.

**Results:**

Parents’ approaches to sexuality education of their boys emerged in six categories: Extreme monitoring and restricting; abstinence as the main content of sexuality education; struggling to establish peace and achieve tolerance; criticizing the cultural taboos; hoping for spontaneous learning; and uncertainty and confusion. The theme “Transition from tradition to modernity” emerged from these categories.

**Conclusion:**

Parents’ approaches to sexuality education ranged from a restrictive traditional manner to approaches with some degree of modern attitudes. Parents are facing uncertainty and confusion regarding sexuality education. Abstinence is an underlying assumption in their sexuality education style. Educating parents through culturally-appropriate methods is a priority that is more acceptable at the policy level. Lack of understanding of the need for sexuality education of children in some parents can threaten the sexual health of children.

## Plain language summary

Children’s sexuality education has an important role in adolescent sexual behaviors and health. However, it is one of the most challenging dimensions of education in families, especially in Muslim communities experiencing the transition to modernity. There is little information about sexuality education of adolescent boys in Iran. In this study we aimed to explore parents’ experiences and perceptions of communicating sexuality education with their sons. Data were collected through individual interview with 11 parents and group discussion with 8 fathers and 8 mothers who had sons aged 10–19, living in Ahvaz, in south west of Iran. We summarized and categorized parents’ approaches to sexuality education in categories including: Extreme monitoring and restricting; abstinence as the main content of sexuality education; struggling to establish peace and achieve tolerance; criticizing the cultural taboos; hoping for spontaneous learning; and uncertainty and confusion. Parents’ approaches to sexuality education ranged from a restrictive traditional manner to some degree of modern conduct. Findings imply that parents are facing challenges regarding sexuality education: uncertainty about the necessity of it, and confusion between religious teachings and modernity. Abstinence is still a central assumption and pre-marital sex is off-limits in their sexuality education. They hoped that they could enhance the children’s adherence to family’s values regarding sexuality, by establishing a peaceful relationship with them. These findings provide evidence for culturally-appropriate educational programs for parents to improve their skills and knowledge about sexuality education. These findings highlighted the fact that Iranian parents are experiencing a “Transition from tradition to modernity”.

## Background

Sexuality education is recognized as a human right and an indispensable necessity for development, but most adolescents have little access to sexuality education, especially those living in developing countries [1]. Notwithstanding, many adolescents engage in high-risk sexual behaviors or sexual abuse that can lead to morbidity and mortality [2].

Several studies have indicated that parent-adolescent sexual communication is associated with lower risky behaviors and more conservative sexual beliefs and attitudes in adolescents [3]. They have also stressed the positive impact of school-based sexuality education [4]. In spite of this, there is immense cultural resistance to sexuality education in Asian cultures. Most parents traditionally believe that talking to children about sexuality can lead to early sexual activity, which is labeled as sexual deviation or unrestrained sex [5, 6]. Misunderstanding of the nature, purpose, and effects of sexuality education can lead to resistance [7]. In the past few decades, a large number of boys have been reported to have no significant communication with their parents on sexual issues and receive most of their sexual information from peers and the media [8]. However, adolescent boys in the United States have been found to have safer sexual behaviors and fewer sex partners, and start sex at an older age due to sexuality education [9].

As a Muslim country experiencing the transition to modernity since two decades ago, Iran has faced serious cultural challenges and major intergenerational conflicts. The socio-cultural challenges have been at the forefront of challenges affecting adolescent sexuality education in Iran [6]. Peers and family have been reported as the first and sixth sources of sexual information for students in Iran, respectively [10]. According to evidence, at least 20% of Iranian adolescents’ experience sexual intercourse [11, 12], and AIDS transmission through unprotected sex has increased dramatically [13]. A recent review in Iran reported that although the majority of young people abstain from premarital sex, high risk sexual behaviors among the sexually active minority are more prevalent in males than females [14].

Studies showed that Iranian parents are confused about what sexuality education is and how it should be performed [14–16]. Studies on sexuality education for female adolescents in Iran indicated that parents face so many challenges in sexuality education for their daughters [6]; adolescent girls, on the other hand, criticized their parents’ conduct in sexuality education [17].

Despite several studies addressing adolescents' sexuality education in Iran, most focused only on girls and there are far fewer studies on boys. This negligence of boys’ sexuality may have cultural roots [18]. A recent study in Iran reports that parents are concerned about their adolescent boys’ sexual behaviors. Another concern is that their children will be sexually abused [19]. While the adolescent boys rated sexual health as the first priority of their health needs [20], more than 80% of Iranian parents have not talked about sexuality with their children [21]. A qualitative study on parents' experiences and perceptions of sexuality education of boys in a Muslim society can help identify basic social processes in this regard. The present study therefor, aimed to elucidate parental strategies adopted for sexuality education of adolescent boys in Iran.

## Methods

This was a qualitative study using conventional qualitative content analysis to explore the Iranian parents’ approaches to their adolescent boys’ sexuality education. The data were collected from January to June 2017. Participants included a total of 27 parents who had adolescent boys aged 10–19 and were from the middle/high social class living in Ahvaz, the capital of Khuzestan province, in southwest of Iran. Participant recruitment and conducting interviews and focus group discussions (FGDs) were done in schools because of the ease of access. Participants were recruited through purposeful sampling with maximum variation. In order to maintain maximum variation, three schools were selected from various city districts characterized with different socioeconomic statuses. Given the cultural sensitivity of the subject of the study and strictness of the Department of Education, official permission was obtained from the Department of Education. After obtaining the consent of the school principal, with the cooperation of the head of the Parents and Teachers Association (who was one of the parents), the parents were informed about the research subject and purpose. Eligible parents who were interested in participating in the study were recruited. The first researcher contacted the volunteer parents and scheduled an interview or FGD. Interviews and FGDs were held at schools by the first researcher-a master’s student of reproductive health. Data collection continued until data saturation. In-depth semi-structured individual interviews were conducted with six fathers and five mothers in a private room. In addition, to providing rich data, two FGDs were held with eight mothers and eight fathers separately. FGD is an Ideal method for data gathering when addressing a sensitive topic [22]. In FGDs, the participants were asked to share their experiences and perspectives about sexuality education of their sons.

The participants were briefed on the study objectives and process, and verbal informed consent was obtained from them. Interviews and FGDs were audio recorded after assuring them of the confidentiality and anonymity of information. The interviews began with a general question (e.g., Please tell me about your experience of your son’s puberty) to allow participants to feel comfortable to start this sensitive subject. Afterward, more specific questions focusing on sexuality education of their adolescent sons were asked (e.g., Tell me about your experience and your methods while providing sexuality education to your son. What is your viewpoint about sexuality education for adolescent boys?) in order to let them feel free to express their experience with sexuality education for their sons. Probing questions and exploratory terms were used to deepen interviews and access much richer data based on the participants’ response (e.g., Would you please explain more about this case by giving examples?). Finally, they were asked to contact the researcher if they had any afterthoughts later. One interview was held for each participant. The interviews lasted from 30 to 70 min, and the length of the two FGDs were 80 and 100 min.

Data collection and data analysis were conducted simultaneously, using methods proposed by Graneheim and Lundman: 1. Verbatim Transcribing all interviews and FGDs immediately after each interview or FGD; 2. Reading the transcripts for several times to understand their overall content; 3. Determining meaning units and primary codes; 4. Classifying similar basic codes in more comprehensive classes, and 5. Determining the latent content contained in data [23].

To enhance the trustworthiness of findings, we followed Lincoln and Guba’s criteria, namely Credibility, Dependability, Confirmability, Transferability and authenticity [24]. To this aim, we took into account these measures: prolonged data collection, purposeful sampling with maximum variation in terms of age and education, member check to validate data and codes, returning of transcripts to participants after coding to ensure the correctness of codes and interpretation, and correcting codes which did not indicate the participants’ intended meaning. Also, peer debriefing and external checks were used too, and codes and categories were reviewed by two experts in qualitative research after initial coding and categorizing by the first researcher. Finally, participants’ quotations were objectively presented.

## Results

The parents were aged from 30 to 53, and their educational attainment varied from high school to master’s degrees. They were either employed by the government or self-employed.

Twenty-four sub-categories, six main categories, and eventually one theme emerged from 73 codes after analyzing parents’ experiences, perceptions, and adopted approaches for the sexuality education of their boys (Table 1).

**Table 1:** Parents’ approaches to sexuality education of adolescent boys

Sub-categories	Categories	Theme
1. Extreme monitoring 2. Restricting access to sexual information 3. Controlling and restricting relationships 4. Keeping silent and dodging	Extreme monitoring and restriction	Transition from tradition to modernity
1. Deviating adolescents’ thoughts from the sexual instinct 2. Attempt to inhibit sexual desire and experiences 3. Resorting to religion as a lever 4. scaring and warning	Abstinence as the main content of sexual education	
1. Avoiding violent behavior 2. Empathy and intimacy with adolescents 3. An optimistic consideration of puberty changes 4. Strict prohibition of sexual behaviors in a gentler manner	Struggling to establish peace and achieve tolerance	
1. Criticism of negative attitudes to sexuality 2. Attempt to desensitize the sexual issues 3. Avoiding extreme restrictions 4. Empowerment instead of restrictions	Criticizing the cultural taboos	
1. Self-modeling for adolescents 2. Metaphorical education 3. Optimism about the adequacy of deterrent boundaries 4. Imitating their own upbringing style	Hope for spontaneous learning	
1. Failure to find information about sexuality education 2. Inefficiency of religious solutions 3. Bewilderment and confusion 4. Disparity in parents’ attitudes toward sexuality education	Uncertainty and confusion	

### Extreme monitoring and restriction

This approach emerged from subcategories including: Extreme monitoring, Restricting access to sexual information, Controlling and restricting relationships, Keeping silent and dodging.

The main approach of most parents engaged in the sexuality education of their sons was to assume a supervisory role and attempt to limit the boys’ access or exposure to sexual information and matters. Parents take controlling measures through visible and invisible monitoring ways in receiving sexual information, controlling and restricting communication, and monitoring the boy’s relationship with peers of the same and opposite sex. Followings are some of the supervisory methods used by parents:

*…We placed the computer in the sitting room where everybody is there if he wants to use it. I put it there so that I can observe him.* (P.32, father, aged 43).

*…When he wants to go to hang out with his friends, I give him the ride so that I can see what kind of people he hangs out with?* (P.2, father, aged 38).

*…We monitor 100%! Both me and his father. We have a satellite at home, but in his presence we do not go as far as possible on channels that may show unrestrained relationships or naked images*. (P5, mother, aged 42).

*…I know that sometimes he watches somethings on his cellphone. His mother has also seen once or twice and rebuked him seriously.* (P.2, father aged 38).

Parents’ reaction to their children’s sexual questions was mostly keeping silent or dodging the issue by referring the boy to another parent, giving unrealistic and childish responses, or postponing the answer until adulthood.

*…It’s really hard. In such cases, I would refer him to his mother. His mother also said that you have to grow up to understand yourself!* (P.4, father, aged 40).

*…We haven’t talked about sexuality. Because he is not a fan of marginal issues! I also never thought he needed to tell him anything about sexual issues* (P.1, mother, aged 45).

Acknowledging the impossibility of adolescent restriction, the parents cited information technology as an important negative factor in sexuality education of children as it makes monitoring and restriction of adolescents difficult for them. They believed that sexuality education is not a responsibility only limited to parents. One of the participants said:

*“In my opinion, there is no way to stop adolescents’ access to sexual information… this is almost impossible. They can do everything by their cellphones, and read and see sexual content in details with photos and videos”* (p.4, father, aged 40).

### Abstinence as the main content of sexuality education

This approach emerged from subcategories including: deviating adolescents' thoughts from the sexual instinct, attempt to inhibit sexual desire and experiences, resorting to religion as a lever, and scaring off and warning.

Most parents tried to deviate their children’s thoughts from sexual issues by forcing them to focus on school or sports activities in an effort to inhibit adolescents’ sexual desire. These parents considered sex before marriage as a sexual deviation promoting promiscuity. Thus they believed that their sons should learn how to abstain until marriage. Most parents try to inhibit the instinctive desires in adolescents, warning them pathogenicity of masturbation, scaring them, and advising on the negative consequences of sexual conduct in adolescence. Parents warned about negative psychological effects of watching pornographic contents and establishing romantic relationships, that can lead to loss of focus on their school subjects and their future. Some parents were also pessimistic about friendship with the opposite sex, and they considered it as an introduction to illegitimate sex. This belief could be found in all of their statements:

*“It’s not just a matter of friendship. I feel they are seeking the real sex and they easily sleep with their girlfriends”*. (P.4, father, aged 40).

*“I completely disagree with relationships between girls and boys at this age, and my son knows if he makes a mistake, how I’m gonna react! When we watch TV series or movies about problems of street friendship, I tell my children to be aware of this, and I warn them that if they have untimely sexual, it will end in the misdirection and even a scandal”.* (P.3, father, aged 43).

Religious parents also resorted to religion in their efforts to inhibit adolescent sexual desires and considered piety as an important factor in adolescent self-control.

*“Nothing like prayer can prevent the youth from going awry. I try to encourage children to pray even by force (he laughs). I do not let them lose their connection with the Quran and mosques. Children are always present in the religious ceremonies of our relatives.*” (P.3, father, aged 43).

### Struggling to establish peace and achieve tolerance

This label was chosen for these subcategories: avoiding violent behavior, empathy and intimacy with adolescents, an optimistic impression of puberty changes, and strict prohibition of sexual behaviors in a gentler manner.

Some parents believed that having empathy with the adolescent, developing a positive and hopeful attitude towards physical/emotional changes of puberty, and avoiding violent behavior could effectively control the sexual behavior in adolescents. In their view, empathy with adolescents builds mutual trust with parents, which is an important factor in adolescent adherence to family values.

*“His mother checked his cellphone and saw that he had exchanged sexual messages and pictures with a girl. We tried to explain logically to him that what he was doing was very wrong. That’s it! I told him that it’s too dangerous and can send both of you to hell. I talked to him more intimately. I saw that he was so scared. I said: “no problem! I understand this. These desires are natural. I was the same as you when I was young”. He apologized and it ended well”*. (P.4, father, aged 40).

### Criticizing the cultural taboos

Criticism of negative attitudes to sexuality, attempt to desensitize the sexual issues, avoiding extreme restrictions, and empowerment instead of restrictions were approaches that we labeled as Criticizing the cultural taboos.

Some parents in this study had positive attitudes towards sexuality and tried to manage this stage of their son’s development and growth by desensitizing sexual issues and considering these behavioral changes as normal. They criticized the negative attitude towards sexuality in the Iranian culture and pointed to its destructive effects on sexual relations in the future of marital life. These parents believed that sexuality should be neither neglected nor suppressed. Rather, instead of imposing radical restrictions on it, sexuality should be approached with a more open mind and a positive attitude. They believe that excessive separation of girls and boys (as is the case in Iran) has adverse consequences. This makes teens more curious to discover and experience sex and experience it much earlier than the appropriate time.

*“Sexual issues are not all negative; they should not be regarded as taboos. This makes the issue more complicated. In Iran, sexual matters have been negatively viewed. The current culture does not provide our kids with the required information and proper training, and because of this, they do not fully enjoy sexual matters. We can emphasize the positive, enjoyable and vital aspects of sexuality”*. (P.4, father, aged 40).

Some parents were well aware of the importance of setting the stage for an appropriate sexuality education as one of the basic prerequisites of the sexual health of their children. For example, by creating intimacy with their children, the parents gave them the courage to express sexual issues, or by cracking jokes, they let their children to talk about private matters.

*“I tell him: O devil! You spent an hour in front of the mirror, do have you a date today? (with a smile). It gives him courage and establishes intimacy little by little. So, if he had a relationship with someone or had any problem, I know what to do, and he can tell me about it”*. (P.2, father, aged 38).

Some parents believed that the desensitization of communication between girls and boys would lead to normalization of sexual desires, which contrary to our expectations, could result in delayed sexual experience in adolescents. Therefore, they allowed the first communication with the opposite sex under parental supervision.

*“I do not want the relationship with the opposite sex to be a taboo or something inaccessible and special for him. That’s too bad because he would think that there must be something special that should be explored on his own. I think that assuming it as a normal issue can help the child to maintain his life the same for more years, and then he can directly experience it”.* (P.5, mother, aged 42).

*“Resistance against this issue is useless. If he goes to military service or university, he will be sexually naive and may immediately fall in love with any girlish coquetry. If I allow my son to experience everything at home, it will have better results and I will have fewer concerns.”* (P.2, father, aged 38).

Admitting the impossibility of a complete restriction of adolescents, especially boys, parents believed that instead of limiting the teenagers’ interactions, they should be empowered to reduce their vulnerability. They considered this as a kind of child immunization to protect them against the dangers of society.

*“I like to make him a bit free; that is, if he experiences some dangerous or inappropriate things, I will consider it as a vaccine that can immunize him against bigger storms!”* (P.9, father, aged 53).

These parents believed that extreme restrictions lead to adolescents' clandestine behavior and cause irreparable harm:

*“Any extreme pressure and restriction will undoubtedly have a reverse effect and makes the satisfaction of this need be done in a much worse and more dangerous way. I’m not against the internet, satellite receivers, cell phones, or even girlfriends, but all of these should be under remote control”.* (P.4, father, aged 40).

### Hope for spontaneous learning

Self-modeling for adolescents, metaphorical education, optimism about the adequacy of deterrent boundaries, and imitating their own upbringing style were approaches that were adopted by some parents.

It is worth mentioning that even some educated parents believed that sexuality education would occur naturally, spontaneously and indirectly through the teenager’s interaction with society.

In fact, the approach some parents adopted for the sexuality upbringing of their sons seemed to depends largely on their own experiences in this regard. They believed that the way they were brought up was positive and so they wished to imitate this method for their sons. A father mentioned it in these words:

*“I have tried to let him gain this information naturally from here and there. I gave him freedom, but I also monitored him. I might be wrong! But I myself learned it (sexuality) from nobody and I gradually learned it from the social environment, especially university. I think this is an appropriate option. It means giving freedom to children in a natural but controlled way; especially nowadays that there are social networks”*. (P.9, father, aged 53).

*I do not think I need to tell him at all. When he grows up, he understands everything himself. It’s better that way. Did anyone teach us? These things should not be said at home.* (P.6, Father, aged 46).

According to the opinion of these parents, in families where moral values prevail and adolescent relationships are controlled, the sexuality education of children is not a major concern, even if they do not directly talk about sexuality education. A father commented:

*“The reason why I don’t get involved in my son’s education in this field is that I’m sure he can do it and overcome this problems thanks to the safe atmosphere in the family, since it had happened to me and posed no problem for me. I have put the responsibility on him to some extent because I knew his personality. His connection with the outside is limited to a few friends, so it is not a concern for me, or at least I am optimistic”* (P.9, father, aged 53).

Emphasizing the fact that parents' behavior serves as a role model for adolescents’ behavior, some parents tried to act as a model for their adolescents, especially during social interactions with the opposite sex:

*“I want my son to follow my lead as much as possible not anyone else. I have seen the impact of this behavior on my son. If I act as a role model myself, I can better impress him”.* (P.4, father, aged 40).

Parents preferred metaphorical and indirect education to direct discussion of sexual issues. For instance, teaching their child to respect individual privacy served as a metaphor for the existence of private relationships. From their point of view, this privacy created a sense of sexual security.

*“We taught him how to respect people’s privacy since childhood; that women and men may have privacy issues. On the other hand, when we knock on the door of his room, this represents showing respect to his dignity and he will feel safe if he has something in his privacy”.* (P.5, mother, aged 42).

### Uncertainty and confusion

This approach emerged from these subcategories: failure to find information about sexuality education, inefficiency of religious solutions, bewilderment and confusion, and disparity in parents' attitudes toward sexuality education.

Most parents felt poorly equipped to have conversation with their sons about sexuality, but were willing to receive information on appropriate approaches of doing it. They pointed out this incompetence with the following statements:

*… I do not know how to tell him! (about sexuality).* (P.6, father, aged 46).

*“Unfortunately, because we are not trained in this field, I do not know what the ideal way is. Should the family play a 100% role? Is it 50–50 between school and family? Is it possible by referring a teenager to read a book? I am almost illiterate in this regard. I have a big question in my mind that what the ideal approach is? Perhaps, there is no a single ideal approach”* (P.9, father, aged 53).

*It’s really weird confusion! I get desperate sometimes! Do I have to quarrel him seriously? Or take her cell phone from him? Or cut the house internet? Or should I go find that girl? In my opinion, these are not the solution. I do not know what to do!* (P.4, father, aged 40).

None of the parents referred to books or any other kind of education for parents about the sexuality education. This issue reflects the neglect of parents’ education in this regard.

*… I have not seen any book in this field* (P.9, father, aged 53).

*… I have tried a lot to find out more about this (sexuality education) but I have not seen*. (P.4, father, aged 40)

parents face the dilemma of following the religious rules or modern life style. They pointed to their confusion between tradition and modernity, to the inefficiency of religious precepts for use nowadays.

*I think 90% of teens masturbate. Well, from a religious point of view, this is a great sin. But what is the solution that religion recommend? What to do? For a young person, sometimes this is the only way to empty himself. In my opinion, any way to suppress this issue fails. Religious says marriage. Should a 16-year-old boy get married? Should he accept the responsibility of a family? It is very different now than it was 1400 years ago!* (P.2, father, aged 38).

Disparity in parents’ approaches to sexuality education was another challenge in families. This means that even in a family, the same attitudes and practice is not adopted by the parents regarding sexuality upbringing.

*I know that sometimes he watches somethings on his cellphone. His mom has seen him watching this stuff and reprimanded him. But I turn a blind eye to it! His mom is too sensitive to these (sexual) issues and makes a big deal of it! Because my wife’s family are very religious and have very restricted relationship. Maybe because I am a man and I understand him more* (P.2, father, aged 38).

*… his father is too strict to these issues (sexuality). If he notices (that he has made a mistake) he will deal severely with him!* (P.7, mother, aged 30).

## Discussion

This study aimed to explore parents’ approaches to sexuality education of adolescent boys in Ahvaz, southwest of Iran. Findings indicated that parents’ approaches to their son’s sexuality education ranged from a lack of understanding the necessity of sexuality education, to acknowledging their ignorance and incompetency for sexuality education. Parents’ experiences and perceptions of sexuality education of their sons reflect their confusion, uncertainty and ineffectiveness in dealing with their children’s sexuality education. Parents struggle with their beliefs, norms of society and their sons’ expectations and beliefs. This confrontation results in a set of conservative policies by parents, enabling them to maintain their power, control performance, reduce the intergeneration gap, and utilize a kind of peaceful upbringing method to decrease tensions and challenges of sexuality education. They adopted an intermediate behavior by relying on their scattered learning influenced by the huge intergeneration gap.

None of the parents referred to books or any kind of education for parents about the sexuality education. This issue reflects the community’s neglect of parents’ education in this regard.

A study in Iran indicated that parents’ beliefs about sexuality upbringing of children are fragilely oscillating. They are not aware of what sexuality education is and how sexuality ethics should be taught. Their approach to sexuality upbringing is a contingency approach. In other words, only when they have a problem, they think about the solution. Until a problem has arisen, they do not explicitly talk about sexual issues, and even sometimes consider it as the reason for attracting adolescents to sexual behavior [15]. This issue was also detected in our study. Some parents, when asked about children’s sexuality upbringing, said that so far there has been no case in which they want to talk to their child about it. Parents ‘refusal to talk to their children about sex can lead to consequences such as adolescents’ pregnancies and STDs that threaten their sexual health.

Studies indicated that while the content of parents’ messages is more focused on abstinence, peer and media messages are significantly about the positivity of sex [8, 25]. Adolescents talk to their peers about the details of sexuality [26]. This is why parents tend to impede their children’s access to permissive attitudes and detailed information about sex by controlling, limiting, and monitoring their children’s relationships with peers.

As mentioned above, parents take efforts to suppress their sons' sexual desire and behaviors by resorting to ways to strengthen religious beliefs and provide warning about sexual behavior. Similarly, other studies reported that Christian parents used church attendance or reading the Bible as ways for their children’s sexual upbringing instead of talking to them directly. Religious parents talk less with their children about sexual relationship or methods of contraception, and they first emphasize religious values and abstinence [27, 28]. According to a study on sexuality upbringing style in the Iranian families, parents paid more (though inadequate) attention to sexual morality, and the least attention was devoted to sexual knowledge [29]. In our study, none of the parents mentioned protected sex in communicating sexuality education with their sons. Parents implied uncertainty about their sons’ commitment to abstinence and raised concern about their engagement in sexual relationship, nevertheless their sexuality upbringing was limited to abstinence, and it is noteworthy that none of the parents referred to skills which enabled teenagers to do abstinence. Even though some of parents referred to the inefficiency of restriction, they rely on it as an appropriate solution. The skill-based sexual health education is supported by international institutions such as WHO, UNESCO, UNFPA in the adolescent sexuality education [1].

One of the significant findings of the present study was the positive attitude of some parents toward sexuality and criticizing sexual taboos in sexuality education. This finding was inconsistent with previous studies conducted on sexuality education of children in Iran over the past few decades. Iranian adolescent girls criticized the negative attitude of adults towards sexuality, and their resorting to frightening and denigration of the consequences of having sex [17]. In fact, socio-cultural changes of Iran in recent decades have changed some parents’ attitude toward the necessity and type of sexuality upbringing of children. Nevertheless, a recent study in Iran has indicated that 82% of parents still do not talk about sexual issues with their children [21]. This is mainly due to lack of parents’ training on how to teach sexual issues. Studies have indicated, On the other hand, studies indicated that there is a relationship between parents’ attitudes and their children’s attitudes toward sexuality. There is also a relationship between parent–child communication about sexuality, and sexual behaviors of children [30]. It means that parents’ negative attitudes toward sexuality can be passed on to their children and parent–child communication about sexuality, can reduce early sexual behaviors of children. This issue emphasizes the importance of educating parents.

There are several opinions highlighting that effective sexuality education should be based on individual values, ethical beliefs, and ethnic and religious backgrounds [31]. Sexuality education will be successful if the cultural context is not ignored [32, 33]. There are some beliefs that what is important in sexuality education is morality. The most important point is that sexual morality is based on family values. Families have both rights and responsibilities in sexuality upbringing [34, 35]. Parents are the most natural sexuality educators for children. They have the best position to support adolescents to face their sexual life [36]. According to our findings, in sexuality education of children, Iranian parents consider themselves entitled to restrict their children’s sexual behaviors rather than being responsible for educating them. This finding highlighted the importance of educating parents.

In our study, some parents showed their optimism about the effectiveness of their monitoring role as well as positive impact of the family’s atmosphere. They considered the unlikelihood of their children’ engagement in sexual relationship, thus they were sure that, firstly, there was little need to directly discuss sexual issues with their teenagers, and secondly, there was no serious threats for their sons. Eisenberg indicated that parents who were sure that their children are engaged in romantic relationships were more likely to talk to their teenagers about sexuality [37]. Previous studies reported an inhibitory impact of parents’ monitoring on adolescents’ sexual behaviors [38].

A comparison of the findings of this study with previous studies indicates that the middle/high-class parents in Iran have relatively similar beliefs about the necessity of abstinence from premarital sex for both their sons and daughters [6]. Similarly, challenges that Iranian parents face in sexuality education are the same for both male and female adolescents. Surprisingly, parents’ concerns about the sexuality education of boys are not less than those of girls, they had the same concerns in this regard [16, 19]. A recent study in Iran showed that parents of adolescent boys have unmet educational needs regarding sexuality education. They believed that both teachers and parents should be trained on sexuality education [19].

At the policy level, despite the fact that some policymakers recognize the importance of addressing adolescents’ sexual health including sexuality education, efforts in this area have been inconclusive due to a lack of consensus on how to provide and what should be the content of this education. Sexuality education in social settings especially for adolescents is facing lack of advocacy [14]. From an Islamic perspective, the relevance of sexuality education in social settings such as schools is controversy because it may violate the principle of modesty. Sexuality education for children does not in itself against modesty. To comply with the principle of modesty, it should preferably be restricted to family environment, and based on Islamic principles i.e., emphasis on abstinence, and prohibition of pre-marital, extra-marital, and homosexuality [34]. However, studies have reported that abstinence-only programs have not been completely successful in preventing risky sexual behaviors in adolescents [39]. Recent studies in some non-Muslim communities have also reported that some parents believe that some issues should only be addressed at home, although they acknowledged that this often does not happen [40].

The present study was among the first studies to address the sexuality education of adolescent boys in Iran. However, it has some limitations: the participants were limited to middle/high class families who were not necessarily representatives of the population. Future studies can enrich data by explaining the adolescents’ experiences of sexuality education they have received from their parents.

### Implications for policy and practice

The findings provide implications for developing adolescents’ sexual health promotion programs in Iran. Focusing on educational programs for parents seems to be a faster and more acceptable solution, given the lack of legal support for formal sexuality education programs in social settings such as schools in Iran. Some parents who acknowledged their lack of competence for sexuality education seems to be open to receiving culture-appropriate educational programs. At the policy level, the life skills approach may be associated with greater acceptance by stakeholders in current political and social conditions of Iran.

## Conclusions

Parents’ approaches to sexuality education of their sons depicts an outlook stream of transition from tradition to modernity. While some parents are still committed to the traditional approaches and optimistic about their effectiveness, others try to desensitize sexuality education by criticizing cultural taboos surrounding sexuality. Findings imply that parents are facing challenges regarding sexuality education: uncertainty about the necessity of it, and confusion between religion teachings and modernity. However, abstinence is still a central assumption in their approaches and pre-marital sex is off-limits in their sexuality education. They hoped that they could enhance the children's adherence to family values, by establishing a peaceful relationship with their children. These findings provide evidence for culturally- sensitive educational interventions for parents to improve their skills and knowledge about sexuality education.

## Data Availability

The datasets used and/or analyzed during the current study are available from the corresponding author on reasonable request.

## Abbreviations

WHO: World health organization
UNFPA: United Nations population fund
UNESCO: United Nations educational, scientific and cultural organization
